# Anton-Babinski Syndrome: A Visual Anosognosia

**DOI:** 10.7759/cureus.55679

**Published:** 2024-03-06

**Authors:** Yasir H Ziaul, Jimmy Mittal, Tazeen Afroze, Vivek Kumar

**Affiliations:** 1 Department of Ophthalmology, TS Misra Medical College and Hospital, Lucknow, IND; 2 Department of Ophthalmology, Vivekananda Polyclinic and Institute of Medical Sciences, Lucknow, IND; 3 Department of Family Medicine, Nad Al Hamar Health Center, Dubai, ARE

**Keywords:** anton-babinski syndrome, visual anosognosia, stroke, posterior cerebral artery, cortical blindness

## Abstract

Anton-Babinski syndrome is a rare extension of cortical blindness following injury to the occipital lobe. The patient behaves as sighted but has visual function derangements. The posterior cerebral artery (PCA) stroke represents 5% to 10% of total strokes. The COVID-19 pandemic has shown a rise in stroke cases. We present a case of this rare PCA stroke, first diagnosed by an ophthalmologist. This case had an inconsistent initial presentation, but subsequent computed tomography of the brain and other neurological investigations confirmed the diagnosis. If such cases are diagnosed early, they could have better management. Timely intervention can decrease morbidity as well as mortality.

## Introduction

A cerebrovascular accident is an injury to the brain secondary to a vascular cause [1]. It is referred to as a transient ischemic attack (TIA) if symptoms last < 24 hours without findings on brain imaging and stroke if symptoms last > 24 hours with findings on brain imaging [1]. The global COVID-19 pandemic has been reported to be linked with neurological disorders, including cerebrovascular accidents [1,2]. The pandemic has shown higher rates of stroke cases in COVID-19-positive as well as in COVID-19-negative patients [1-5]. Ischemic stroke was a more common manifestation than hemorrhages [1]. COVID-19 patients had a 3.6 times increased risk of ischemic stroke compared to non-COVID-19 patients [4]. COVID-19 may progress asymptomatically without getting detected [5]. Viral loads of asymptomatic patients were found to be significantly higher than symptomatic patients [6]. Even mild COVID-19 symptoms were associated with a 1% risk of stroke [4]. High levels of inflammation, hypercoagulability, and/or medical severity predispose to thromboembolism formation [4-6]. This can increase the risk of in situ thrombosis and embolus from distant parts can also travel to the brain, leading to ischemia and infarction [4]. The most common source of embolus in ischemic occipital lobe infarction is from cardiac origin [1]. The occipital cortex is especially sensitive to hypoxia because of its relatively distal location from the central cerebral vasculature [7]. Occlusion of specific vessels of the brain by thrombus or embolus may lead to infarction and their clinical presentation varies according to the location and area covered by the affected vessel [1].

Cortical blindness (CB) is vision loss due to insult to unilateral or bilateral lesions of the occipital cortex with intact anterior visual pathways [7]. In most cases, it is binocular with a normal papillary reaction [8]. Anton-Babinski syndrome (ABS or Anton syndrome) comprises an obvious vision loss due to CB, associated with visual anosognosia (denial of loss of vision) and confabulation [7,8]. Confabulation is the emergence of memories of events and experiences that never took place [7]. Patients behave to have normal vision with the help of confabulation and description of their surroundings [7]. The patient will continue to deny that they cannot see but the defect becomes apparent when patients walk into objects and are found describing people or surroundings that are not present [8]. Here, we present a case of ABS during the COVID-19 pandemic with a negative COVID-19 report to insist on a thorough examination even for a minor atypical visual symptom that could prevent morbidity and even mortality due to accident.

## Case presentation

A 48-year-old male farmer experienced sudden onset blurring of vision in both eyes after lunch. He fell to the ground and became unconscious for a few minutes. It was not associated with symptoms of nausea, vomiting, headache, or seizure. After regaining consciousness, he could perform daily living activities with minimum assistance but he was bumping into objects while walking. So, family members took him to a hospital the next day.

The patient complained of a moderate headache without fever. The patient was thin-built and without a history of diabetes mellitus, hypertension, heart disease, trauma to the head, or COVID-19 infection. He had no habits of nicotine abuse or alcohol consumption at the time of the fall. Glasgow Coma Scale (GCS) score was 15 out of 15 and he had normal power in all four limbs with no sensory loss. Blood pressure was 130/90 mmHg and random blood sugar was 96 mg/dl. He tested COVID-19 negative and was not vaccinated for COVID-19 at that time.

On examination, visual acuity was severely impaired, but the patient insisted that he could see things present around him. When asked, it was difficult for him to identify items present in the surroundings, but color perception was present. It was easier for him to identify moving things than static ones. There was no systemic association for dizziness, dysarthria, limb weakness, gait ataxia, or nystagmus.

**Figure 1:**
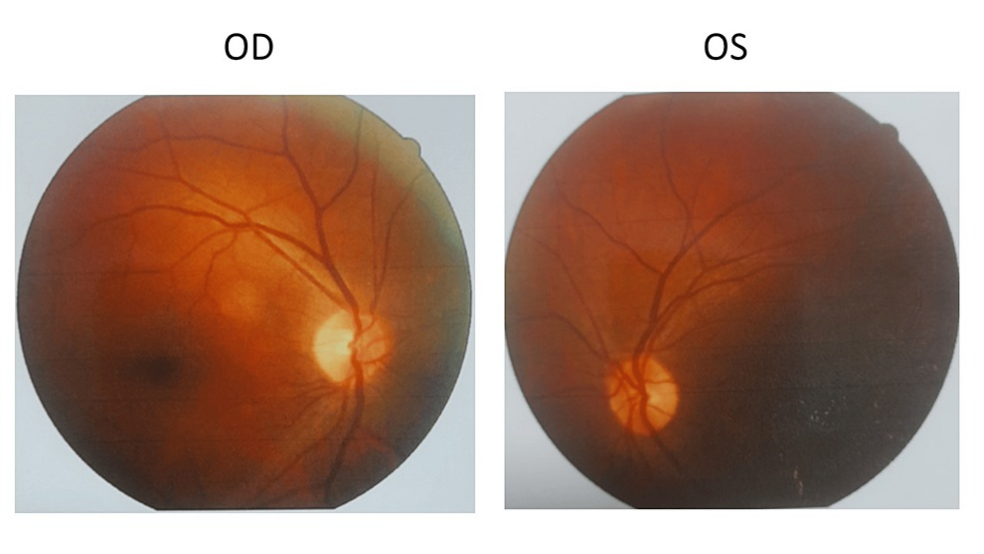
Retinal image of both eyes of the patient with Anton-Babinski syndrome. The optic nerve head, vessels, macula, and peripheral retina are normal. OD: right eye; OS: left eye.

Visual acuity was hand movement (HM) in both eyes with the projection of light perceived from all cardinal directions. Color vision (CV) was normal. On examination, the face was bilaterally symmetrical. Ocular adnexa was normal. The extraocular movement was within normal limits. Pupillary reflexes were normal and brisk. Slit lamp biomicroscopic examination (Topcon, Tokyo, Japan) revealed early lenticular changes but the posterior segment of the eyes was normal. Fundus photography was done using the Canon DRC CX-1 camera (Canon Medical Systems, Tustin, CA) (Figure 1).

**Figure 2:**
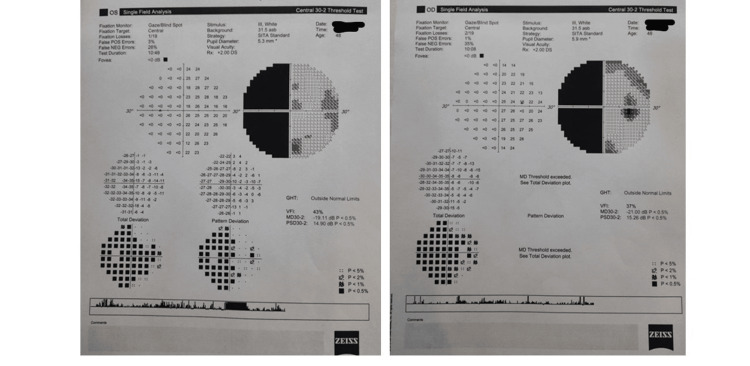
Field of vision chart of both eyes of the patient with Anton-Babinski syndrome. Field analysis central 30/2 shows left homonymous hemianopia (congruous).

Intraocular pressure was 12 mmHg (Reichert 7 Non-Contact Tonometer, Reichert, Inc., Depew, NY) in both eyes. We were unable to perform optokinetic nystagmus and visual response to threat. Visual field (VF) (Zeiss Humphrey Field Analyzer 3, Baden-Württemberg, Germany) central 30/2 showed left congruous homonymous hemianopia (HH) (Figure 2).

**Figure 3:**
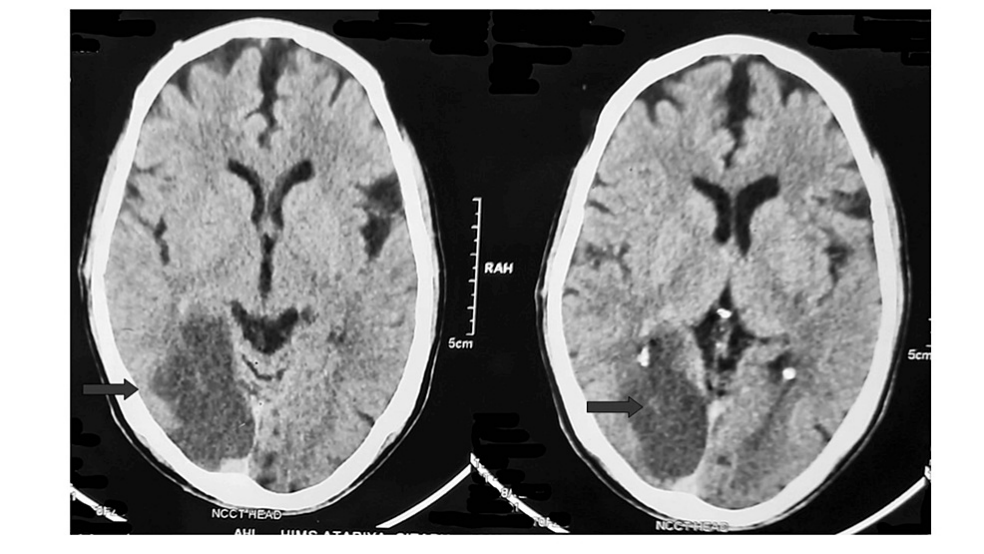
Computed tomography of the brain and skull of the patient with Anton-Babinski syndrome. Sections of the brain in CT scan. Arrows show an infarct area in the right occipital lobe.

The patient was urgently referred for further neurological evaluation. Brain non-contrast CT (NCCT) was done that found ill-defined hypodensities in the right temporo-occipital lobe suggestive of ischemic infarct in the right posterior cerebral artery (PCA) territory (Figure 3).

The patient was treated with a nerve-protecting agent, gamma-aminobutyric acid (GABA) analog, nutritional supplement, and analgesic for two months. The patient is under follow-up for four months without signs of visual recovery.

## Discussion

Atypical visual loss in a patient should raise suspicion and consideration of CB and ABS [8]. The etiology of CB includes cerebrovascular disease due to stroke, thrombosis, embolus, eclampsia, etc. [7]. Cerebrovascular disease causing ischemia of the occipital cortex is the most common pathology of CB and ABS [7,8]. The occipital cortex is far from the central vasculature in the brain, so it is sensitive to hampered oxygen supply or ischemia [7]. This insult within the brain leads to a visual defect known as neurological visual impairment, which includes CB, ABS, HH, visual neglect, visual agnosia, etc. [8].

In our case, the patient was able to detect moving objects more easily than static ones and CV was normal. The CV and detection of motion are governed by the V5 area of the visual cortex [9]. Some subcortical fibers bypass the V1 area of the primary visual cortex and connect directly to the V4 and V5 areas of the secondary visual cortex [9]. This could be a reason for normal CV and detection of moving objects in our case.

VF showed left congruous HH in our case. HH is the most common cortical visual impairment, leading to blindness in the contralesional hemifield [10-12]. It supports retrochiasmal pathology [11]. Stroke patients involving the V1 primary visual cortex area were found to have HH (30%), and it was mostly congruent [9,12]. These HH patients were supposed to be blind in half contralesional VF but some patients present residual capacities in that blind area known as blindsight [12]. Similarly, the seeing area of ipsilateral VF in these patients may show a subtle deficit, known as sightblindness [9,12]. Sightblindness is just the reverse of blindsight [12].

NCCT confirms an ischemic infarct in the right temporo-occipital lobe. PCA supplies the temporo-occipital lobe [11]. Among strokes, only 5-10% are represented by PCA stroke [12]. The PCA travels from the deep to the superficial area of the brain, which can be divided into the proximal (deep) part (P1 and P2 segments) and the distal (superficial) part (P3 and P4 segments) [11,12]. P4 segment pathology leads to VF defects [11]. Patients may have only a headache and mild visual changes [12]. This may delay getting medical treatment as the patient may consider it to be a minor disease and critical time of the intervention will pass out (as in our case) [11].

The COVID-19 pandemic has shown a rise in ischemic stroke cases [5,12]. Asymptomatic patients may also have a high viral load in the blood [6]. These high viral loads in the blood may produce changes in the structure of the vascular wall as well as increase the coagulation in the vascular lumen [5,12], leading to complications like stroke and a source of infection for others without getting detected [5]. Our patient had a moderate headache with visual symptoms, and he being an asymptomatic carrier cannot be ruled out.

ABS is an emergency, just like a stroke. The most important aspect of treatment is the time of onset of symptoms [11]. It will decide the mode of treatment required in an acute setting, for the etiology of occipital lobe damage. If the patient reaches within 4.5 hours of being symptomatic, then intravenous tissue plasminogen activator (tPA) has a role; otherwise, for those presenting late (after 4.5 hours), as in our case, the treatment is focused on preventing further stroke and rehabilitation [12,13]. Quick investigation to find out the etiology and an anticoagulant or antiplatelet is administered to prevent further stroke [11,12]. Other risk factors, like hypertension, cholesterol, diabetes, etc., should be addressed [11-13].

## Conclusions

We present this case to create awareness as well as to add to the limited literature on ABS. The occipital lobes were affected in our case due to cerebral vascular accidents. We put forward the importance of meticulous history taking, complete ophthalmic examination with collaborated teamwork, including a neurologist and radiologist, and intervention without delay, as treatment depends upon the timing of the onset of symptoms. The prognosis depends upon etiology, age, severity, time since onset, any initial recovery present, and associated medical history. In our case, a poor visual prognosis may be his late arrival for treatment. If such cases are diagnosed early, they could have better management outcomes and morbidity as well as mortality could be decreased.
